# Intervention Enhancement Strategies Among Adults With Type 2 Diabetes in a Very Low–Carbohydrate Web-Based Program: Evaluating the Impact With a Randomized Trial

**DOI:** 10.2196/15835

**Published:** 2020-09-09

**Authors:** Laura R Saslow, Judith Tedlie Moskowitz, Ashley E Mason, Jennifer Daubenmier, Bradley Liestenfeltz, Amanda L Missel, Hovig Bayandorian, James E Aikens, Sarah Kim, Frederick M Hecht

**Affiliations:** 1 Department of Health Behavior and Biological Sciences, School of Nursing University of Michigan Ann Arbor, MI United States; 2 Northwestern Medicine Feinberg School of Medicine Chicago, IL United States; 3 Osher Center for Integrative Medicine University of California San Francisco San Francisco, CA United States; 4 Department of Health Education San Francisco State University San Francisco, CA United States; 5 Department of Family Medicine University of Michigan Ann Arbor, MI United States; 6 Division of Endocrinology, Diabetes and Metabolism University of California, San Francisco San Francisco, CA United States

**Keywords:** type 2 diabetes, diet, ketogenic, text messages, self-management

## Abstract

**Background:**

Adults with type 2 diabetes may experience health benefits, including glycemic control and weight loss, from following a very low–carbohydrate, ketogenic (VLC) diet. However, it is unclear which ancillary strategies may enhance these effects.

**Objective:**

This pilot study aims to estimate the effect sizes of 3 intervention enhancement strategies (text messages, gifts, and breath vs urine ketone self-monitoring) that may improve outcomes of a 12-month web-based *ad libitum* VLC diet and lifestyle intervention for adults with type 2 diabetes. The primary intervention also included other components to improve adherence and well-being, including positive affect and mindfulness as well as coaching.

**Methods:**

Overweight or obese adults (n=44; BMI 25-45 kg/m^2^) with type 2 diabetes (glycated hemoglobin [HbA_1c_] ≥6.5%), who had been prescribed either no glucose-lowering medications or metformin alone, participated in a 12-month web-based intervention. Using a 2×2×2 randomized factorial design, we compared 3 enhancement strategies: (1) near-daily text messages about the intervention’s recommended behaviors (texts n=22 vs no texts n=22), (2) mailed gifts of diet-relevant foods and cookbooks (6 rounds of mailed gifts n=21 vs no gifts n=23), and (3) urine- or breath-based ketone self-monitoring (urine n=21 vs breath n=23). We assessed HbA_1c_ and weight at baseline and at 4, 8, and 12 months. We evaluated whether each strategy exerted a differential impact on HbA_1c_ and weight at 12 months against an a priori threshold of Cohen *d* of 0.5 or greater.

**Results:**

We retained 73% (32/44) of the participants at 12 months. The intervention, across all conditions, led to improvements in glucose control and reductions in body weight at the 12-month follow-up. In intent-to-treat (ITT) analyses, the mean HbA_1c_ reduction was 1.0% (SD 1.6) and the mean weight reduction was 5.3% (SD 6.0), whereas among study completers, these reductions were 1.2% (SD 1.7) and 6.3% (SD 6.4), respectively, all with a *P* value of less than .001. In ITT analyses, no enhancement strategy met the effect size threshold. Considering only study completers, 2 strategies showed a differential effect size of at least a *d* value of 0.5 or greater

**Conclusions:**

Text messages, gifts of food and cookbooks, and urine-based ketone self-monitoring may potentially enhance the glycemic or weight loss benefits of a web-based VLC diet and lifestyle intervention for individuals with type 2 diabetes. Future research could investigate other enhancement strategies to help create even more effective solutions for the treatment of type 2 diabetes.

**Trial Registration:**

ClinicalTrials.gov NCT02676648; http://clinicaltrials.gov/ct2/show/NCT02676648

## Introduction

Type 2 diabetes is a costly [1] and deadly illness [2] affecting more than 30 million Americans. Our previous research suggests that a web-based *ad libitum* very low–carbohydrate, ketogenic diet (VLC) and lifestyle intervention that includes training in positive emotions and mindful eating can help overweight adults with type 2 diabetes improve their blood glucose control and lose weight [3,4]. This, in turn, may reduce the future risk of health complications [5]. Although other approaches, such as very low–calorie diets, may also increase glycemic control and reduce the need for antidiabetic and antihypertensive drugs [6], recent recommendations from the American Diabetes Association [7] and other reviews of research and clinical evidence [8,9] support the use of very low–carbohydrate diet interventions.

In this study, we evaluated 3 potentially helpful enhancements to the VLC diet and lifestyle web-based intervention: (1) text messages, (2) food and book gifts, and (3) type of dietary adherence self-monitoring using different measures of ketones (urine vs breath). Although potentially beneficial, these enhancements may also increase participant burden or increase intervention costs. Thus, the primary goal of this pilot study was to determine which methods may be effective for enhancing behavior change in future trials. All participants received access to a comprehensive web-based intervention that included positive emotion and mindful eating training in addition to dietary guidelines [4]. We then varied, in a 2×2×2 full factorial design, whether participants received each of the 3 extra intervention enhancements or not.

First, we varied whether or not participants received text messages targeting improved intervention adherence. Message-based interventions have been shown to improve a wide variety of health behaviors, possibly because of their ability to remind participants of intervention-relevant behaviors or to address barriers to adherence [10,11]. Messages based on this particular diet and lifestyle intervention have not been previously tested. However, messages might also increase intervention costs and complexity and/or burden participants. Thus, this strategy should be tested before being included in future interventions.

Second, we tested the impact of mailing gifts of diet-relevant foods and cookbooks (6 rounds of mailed gifts vs none). Although providing meal replacements has generally been found to be helpful for weight loss [12,13], to our knowledge, it is novel to provide gifts of intervention-related foods and cookbooks. Previous research has hypothesized that mailed gifts increase positive affect, which, in turn, may increase intervention adherence [14]. However, this approach likewise adds expense, approximately US $150 per participant in our design, and thus should be carefully evaluated for inclusion in future interventions.

Third, we assessed the impact of urine versus breath ketone self-monitoring. Self-monitoring may increase behavioral adherence to dietary interventions by providing external feedback for the targeted behaviors [15,16]. Such self-monitoring behavior improves diabetes self-management [17], and greater dietary self-monitoring is generally related to greater weight loss and dietary adherence [18]. People adhering to a VLC diet should produce ketones detectable in the breath or urine [19]. Hence, we sought to help participants self-monitor their ketones and thus dietary adherence in a less burdensome way than tracking their diet directly because dietary self-monitoring can be burdensome and long-term daily adherence can degrade in the long term [20]. We were especially interested in testing this enhancement strategy because of the price difference between the 2 ketone measurement approaches: when we conducted this trial, the urine test strips cost approximately US $25 for 100 strips, and the breath meter costs approximately US $150. We sought to determine whether the more expensive method of monitoring dietary adherence was actually more beneficial for participants.

Our study design was informed by the multiphase optimization strategy, which encourages intervention optimization through full factorial designs, allowing us to efficiently identify promising enhancement strategies for more definitive future testing. By using a full factorial as opposed to a three-arm study, this design requires fewer participants to rule in or out potentially promising intervention strategies [21]. The overriding goal of this trial was to help inform decisions about which enhancement strategies may be the most promising and should be combined into a treatment package to be tested in a full-scale follow-up trial [22].

## Methods

### Procedure

The institutional review board (IRB) at the University of Michigan, which also served as the IRB of record for study investigators at the University of California, San Francisco, approved the study materials (HUM00102827). We registered this study with clinicaltrials.gov (NCT02676648). We recruited participants between February 2016 and November 2016 and completed data collection by October 2017. We placed advertisements or notices of the research on the web (including Reddit, Facebook, Craigslist, University of Michigan’s web-based portal for clinical trials, LinkedIn, Pandora radio, and ResearchMatch) and sent invitation letters to potentially eligible participants identified from health plan records at Michigan Medicine. We directed interested participants to the study website, where they completed a web-based self-report screening survey (Qualtrics) and where we displayed the logos of both schools involved. Those who were eligible for further screening based on their survey responses were asked to provide web-based electronic consent to undergo a second web-based survey (Qualtrics); self-administered glycated hemoglobin (HbA_1c_) test from DTI Laboratories, Inc; and 3 days of dietary tracking on MyFitnessPal. We also mailed these individuals a body weight scale that connects to its own cellular network (BodyTrace). Finally, those who met all entry criteria (below) were invited to participate in the trial.

Participants were eligible to participate if they were aged 21-70 years, had a current HbA_1c_ of 6.5% or higher (measured with the at-home test), had a BMI of 25 to 45 kg/m^2^ (based on self-reported height and measured weight per electronic communication from the mailed scale), had access to the internet for personal use, were willing to check their email at least once a week, were comfortable reading and writing in English, had no potentially serious comorbidities such as liver or kidney failure, were planning on living in the United States for the duration of the trial, were not vegetarian or vegan, and were not on medications known to cause weight gain such as second-generation antipsychotics. Given that this study was conducted remotely, to mitigate the risk of hypoglycemia, we excluded participants who reported taking any glucose-lowering medication other than metformin.

### Experimental Design

This 2×2×2 full factorial experiment examined the impact of 3 experimental, two-level factors. We randomized the participants to 1 of 8 unique experimental conditions (Table 1).

Once all baseline measurements had been completed, the study staff randomized the participants to one of the abovementioned 8 conditions using a computer program to reveal the next assignment. The order was created using block randomization procedures, with blocks randomly allocated to size 8 or 16 and with the seed number of 714119960524911 from the Sealed Envelope website, we create a blocked randomization list [23].

**Table 1:** All tested combinations of the 3 intervention enhancement strategies.

Combinations	Experimental factors		
	Texting	Gifts	Ketone measurement
1	Texts	Gifts	Urine
2	Texts	Gifts	Breath
3	Texts	No gifts	Urine
4	Texts	No gifts	Breath
5	No texts	Gifts	Urine
6	No texts	Gifts	Breath
7	No texts	No gifts	Urine
8	No texts	No gifts	Breath

#### Standard Intervention

We encouraged all participants to eat an *ad libitum* (noncalorie-restricted) VLC diet, as in our previous research [3,4], which focused on reducing carbohydrate intake to between 20 and 35 nonfiber grams a day and including calories derived from meats, cheeses, dairy products, eggs, fats, nuts, seeds, and low-carbohydrate vegetables and fruits. If participants experienced muscle cramps, we suggested that they consider taking over-the-counter magnesium supplements as needed.

We also provided participants with strategies to increase day-to-day positive emotions, mindfulness, and mindful eating [24-28]; a coach (the first author), who answered the participants’ questions via email or phone [29]; encouragement to be physically active [30] and get sufficient sleep [31]; information about web-based VLC support groups; and suggestions to track their diet using a free web-based and mobile app, MyFitnessPal [32], daily in the first month, and starting in the second month, for 3 consecutive days every 4 weeks. We did not test basic aspects of the intervention, such as weekly emails and access to an email-based coach, via a full factorial trial design in this study. This is because such a trial design is best suited to testing potential components that may be costly or burdensome to participants.

The intervention lasted 12 months. During the first 4 months, we emailed the participants weekly. These 16 emails contained links that connected them to (1) a short survey to assess intervention-related adherence and health concerns; (2) a short, embedded video to teach assigned topics; (3) downloadable handouts to accompany the videos; and (4) links to external resources pertaining to that week’s information. As some participants may prefer not to watch videos, we also provided video transcripts in a downloadable PDF format. Participants could watch and read the lessons whenever they wished. Lessons varied in length but, on average, required approximately 10 to 30 min to complete, including watching the video and reading the handouts. For the remaining 8 months of the program, we emailed participants links to the coursework every other week.

#### Three Experimental Enhancement Strategies

Once we assigned participants to 1 of 2 levels of each factor, we sent them assignment-specific materials throughout the 12-month intervention.

#### Text Messages

To encourage the adoption, engagement, and maintenance of the intervention, we randomized half of the participants to receive an average of 5 (SD 0.2) text messages per week (5 sent for 50 weeks, 6 sent for 2 of the weeks, sent each day between 9 AM and 5 PM). These were drawn from a pool of 262 unique messages that included motivational and educational reminders about the intervention’s lessons or goals, advice about the VLC diet (recipes, web-based resources, and quotes from others who had tried the diet), advice about physical activity (with an emphasis on finding activities they enjoyed), advice about sleep (such as suggestions about sleep hygiene behaviors), and advice about psychological skills (around positive emotions, mindfulness, and mindful eating). The other half of the participants received no text messages.

#### Food and Book Gifts

We randomly assigned half of the participants to receive a mailed assortment of unusual and hard-to-find foods relating to the VLC diet and popular lay-press cookbooks or books specifically tailored for or about the diet. At baseline, we mailed these participants an assortment of foods for the VLC diet: 1 pound each of almond flour, coconut flour, and chia seeds as well as 1 ounce of liquid sucralose. They also received popular lay-press cookbooks or books specifically tailored for or about the diet at different times throughout the 12-month program (at baseline: *Keto Living 3 Cookbook: Lose Weight with 101 All New Delicious and Low Carb Ketogenic Recipes* [33]; at 3 months: *Bacon & Butter, the Ultimate Ketogenic Diet Cookbook* [34]; at 5 months: *The Wicked Good Ketogenic Diet Cookbook: Easy, Whole Food Keto Recipes for Any Budget* [35]; at 7 months: *Good Calories, Bad Calories: Fats, Carbs, and the Controversial Science of Diet and Health* [36]; at 9 months: *The KetoDiet Cookbook: More Than 150 Delicious Low-Carb, High-Fat Recipes for Maximum Weight Loss and Improved Health* [37]; and at 11 months: *Quick & Easy Ketogenic Cooking: Meal Plans and Time Saving Paleo Recipes to Inspire Health and Shed Weight* [38]). If participants in this group told study staff that they had difficulty with VLC adherence, we mailed them supplemental books and/or food products. This occurred with 2 participants. One participant was mailed several types of commercially available VLC breads. Another, whose computer was temporarily not functional, was mailed a physical copy of *The Ketogenic Diet: A Scientifically Proven Approach to Fast, Healthy Weight Loss* [39]. Participants in the nongifts group were not sent anything extra.

#### Urine Strips Versus Breath Meter for Ketone Self-Monitoring

We randomly assigned participants to self-monitor their dietary adherence biomarkers using either a urine- or breath-based meter. The urinary ketone test kits (KetoStix, Abbott; included 100 strips) provide feedback about urinary ketone acetoacetate. The breath meter (Breath Ketone Analyzer, Ketonix) measures the exhaled ketone acetone. We asked participants to use these at least once weekly for the first few months of the intervention.

### Assessments

We measured outcomes at baseline and at 4, 8, and 12 months after baseline. As an incentive for continued participation, we paid participants US $25 for completing their outcome measurements at 4 months, US $25 at 8 months, and US $50 at 12 months. At each period, we measured glycemic control and body weight (described below), and using web-based surveys at Qualtrics, we assessed the perceived helpfulness of the enhancement strategies (rated from 1 [*not helpful*] to 7 [*very helpful*]) and overall program satisfaction (rated from 1 [*not at satisfied*] to 7 [*very satisfied*]). For this particular study design, it was impossible for the participants or coach to be masked to the allocation status.

#### Glycemic Control

We assessed glycemic control by measuring HbA_1c_ using the self-administered, mailed AccuBase HbA_1c_ test (DTI Laboratories). This Food and Drug Administration–approved whole blood test uses a capillary tube blood collection method for reliable home-based data collection and high-performance liquid chromatography laboratory testing.

#### Body Weight

We measured body weight by mailing participants a scale that connects to its own cellular network. This method corresponds well to same-day in-person measurement by research staff [40] and has a back-end interface to allow easy download of participant data. As it connects via its own cellular phone network, participants do not have to set up any passwords, simplifying ease of use. We encouraged participants to weigh themselves weekly but only requested it at baseline and at 4, 8, and 12 months postbaseline. To ensure that we measured the participants’ weight and not someone else’s in their household who was using the scale casually, for these critical measurements, we asked them to step twice on the scale within 5 min. We averaged the 2 measurements to estimate their weight.

### Analytic Plan

For completers-only analyses, we excluded participants who did not complete the 12-month assessment. For intent-to-treat (ITT) analyses (all participants included), we imputed missing 12-month values using the last observation carried forward method, one option for handling missing data in clinical trials [41]. We first collapsed across all groups and examined pre-post 12-month changes in HbA_1c_ and percent weight change using within-subjects *t* tests. We then examined the effect sizes of the 2 levels of each enhancement strategy compared with one another using Cohen *d* (using a pooled SD of the 2 levels of each strategy). Our primary goal was to screen each strategy for a medium effect size represented by an a priori Cohen *d* threshold of 0.5 [42]. Moreover, because all participants were assigned to an active intervention and our sample size was small, we understood that we may not reach statistically significant differences between the levels of the enhancement strategies. However, we focused on effect size differences, as nonsignificant between-level effect sizes can still help advise which enhancement strategies may be worth including in future trials. At 12 months, we also examined participants’ impressions of the strategies and whether the strategies altered their overall satisfaction with the program (comparing the groups with *t* tests).

## Results

**Figure 1:**
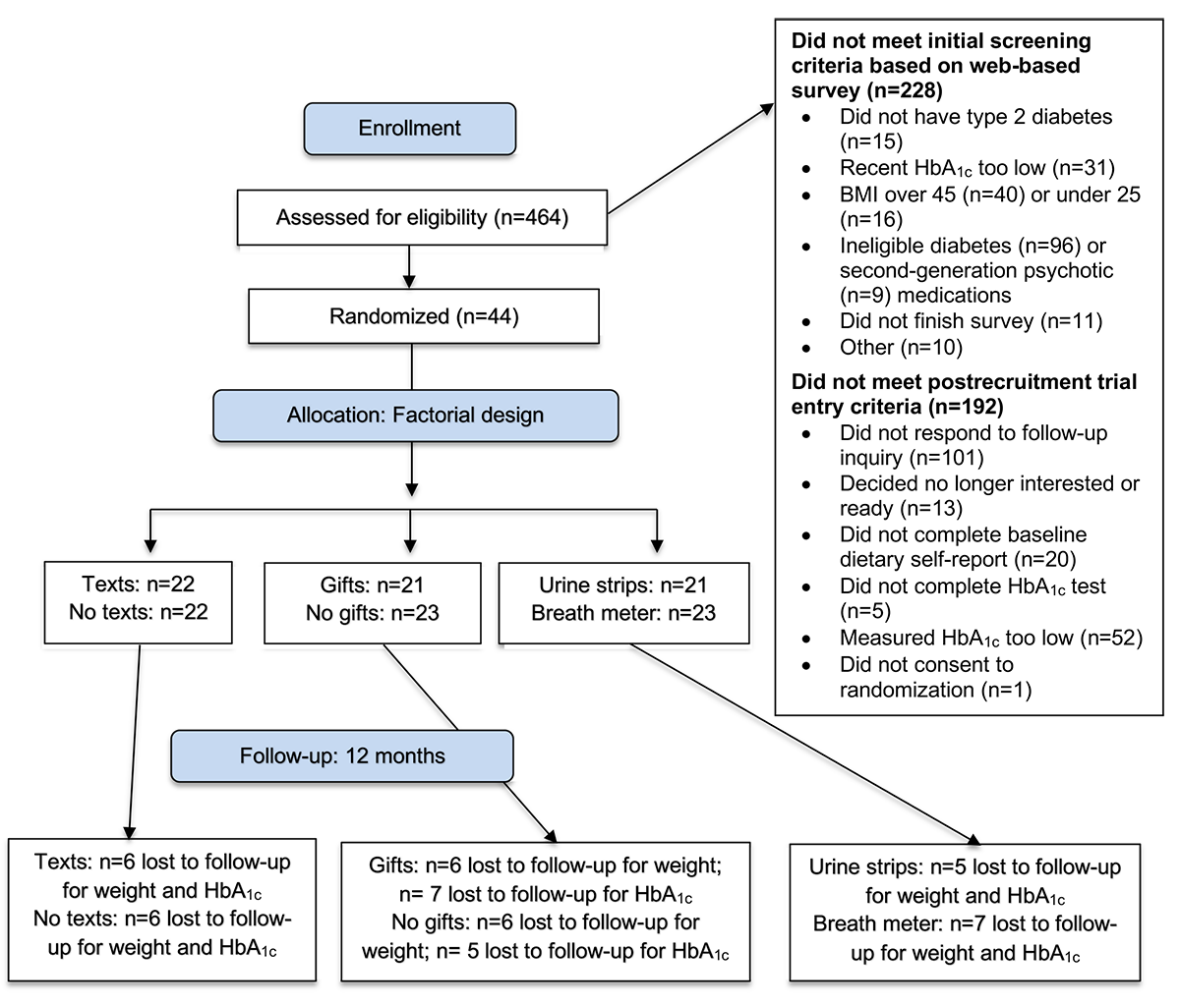
Study participant ﬂowchart. HbA_1c_: glycated hemoglobin.

We screened a total of 464 potential participants. We excluded potential participants if they used hypoglycemic medications other than metformin (n=96), reported a recent HbA_1c_ below 6.5% (n=31), had a measured HbA_1c_ below 6.5% (n=52), self-reported BMI above 45 kg/m^2^ (n=40), or did not provide usable contact information in the original survey (n=101; Figure 1).

Ultimately, we enrolled and randomized 44 participants, who were, on average, aged 52 years, had diagnosed type 2 diabetes for about 5 years, and started with an HbA_1c_ of 8.4% (Table 2). Approximately half of the participants were randomized to each level of the 3 experimental components (Figure 1). All participants lived in the United States, and half of the participants lived in Michigan.

**Table 2:** Baseline participant characteristics (n=44).

Characteristics			Participants
**Sex, n (%)**			
	Men	11 (25)	
	Women	33 (75)	
Age (years), mean (SD)			51.7 (11.0)
**Race or ethnicity, n (%)**			
	American Indian or Alaska Native	1 (2)	
	Asian or Pacific Islander	5 (11)	
	Black	7 (16)	
	White	33 (75)	
	Latino or Latina	0 (0)	
Duration of diabetes (years), mean (SD)			5.3 (4.1)
Smoker, n (%)			2 (4)
HbA_1c_^a^ (%), mean (SD)			8.4 (2.2)
Weight (kg), mean (SD)			100.2 (20.1)
BMI (kg/m^2^) mean (SD)			35.7 (5.6)
College graduate, n (%)			27 (61)
Married or long-term partner, n (%)			22 (50)
**Total household income (US** **$),** **n (%)**			
	≤35,000	13 (29)	
	35,001-75,000	18 (41)	
	≥75,001	12 (27)	

^a^HbA_1c_: glycated hemoglobin.

We retained 73% (32/44) of the participants at 12 months. For HbA_1c_, of the 11 participants who lacked a 12-month follow-up, month 8 data were carried forward for 4 participants, month 4 data were carried forward for 5 participants, and baseline data were carried forward for 2 participants. For weight, of the 12 participants who lacked a 12-month follow-up, month 8 data were carried forward for 4 participants, month 4 data were carried forward for 6 participants, and baseline data were carried forward for 2 participants.

The VLC web-based multicomponent intervention, across all conditions, led to improvements in glucose control and body weight at 12-month follow-up. In the ITT analyses (including all participants), the mean HbA_1c_ decreased by 1.0%, and the mean weight was reduced by 5.3% (*P*<.001). Overall, 27% participants (12/44) achieved excellent control of their type 2 diabetes (HbA_1c_<6.5%), 43% participants (19/44) lost at least 5% of their body weight, and 23% participants (10/44) lost at least 10% of their body weight. For study completers, the mean HbA_1c_ was reduced by 1.2%, and the weight was reduced by 6.3% (*P*s<.001). Of completers, 31% (10/32) achieved excellent control of their type 2 diabetes (HbA_1c_<6.5%), 47% participants (15/32) lost at least 5% of their body weight, and 31% participants (10/32) lost at least 10% of their body weight.

In ITT and completers-only analyses, none of the extra enhancement strategies exerted a statistically significant impact on either HbA_1c_ or weight (all *P*>.10). Among study completers, 2 enhancement strategies met our a priori threshold of Cohen *d* of 0.5 or greater for differential effect sizes: text messages (vs no text messages) for HbA_1c_ reduction and urine ketone self-monitoring (vs breath ketone self-monitoring) for weight reduction (Table 3). None of the enhancement strategies met our Cohen *d* threshold in ITT analyses. Although the effect size for gifts did not meet our a priori threshold, it did have a small effect size, as Cohen *d* ranged from 0.2 to 0.3.

**Table 3:** Change in outcomes over 12 months.

Variable		Absolute HbA_1c_ change relative to baseline				Percent weight change relative to baseline				
		All (ITT^a^)	*P* value	Completers	*P* value		All (ITT)	*P* value	Completers	*P* value
**Overall**										
	Change (%), mean (SD)	−0.98 (1.58)	.001	−1.20 (1.65)	.001		−5.25 (6.04)	<.001	−6.32 (6.36)	<.001
	Cohen *d*	−0.35	–	−0.56	–		−.27	–	−0.48	–
**Text messages**										
	Yes (%), mean (SD)	−1.24 (1.89)	–	−1.59 (1.91)	–		−5.44 (6.70)	–	−6.95 (7.13)	–
	No (%), mean (SD)	−0.74 (1.24)	–	−0.85 (1.35)	–		−5.08 (5.52)	–	−5.76 (5.76)	–
	Difference (%), mean (SD)	−0.50 (0.48)	.30	−0.75 (0.58)	.21		−0.36 (1.84)	.85	−1.19 (2.28)	.61
	Cohen *d*	−0.32	–	−0.46	–		−0.06	–	−0.18	–
**Gifts**										
	Yes (%), mean (SD)	−1.14 (1.60)	–	−1.41 (1.88)	–		−5.78 (6.80)	–	−7.26 (7.08)	–
	No (%), mean (SD)	−0.83 (1.59)	–	−1.03 (1.48)	–		−4.77 (5.37)	–	−5.48 (5.74)	–
	Difference (%), mean (SD)	−0.31 (0.48)	.53	−0.39 (0.59)	.52		−1.01 (1.84)	.59	−1.79 (2.27)	.44
	Cohen *d*	−0.19	–	−0.23	–		−0.17	–	−0.28	–
**Ketone measurement**										
	Urine (%), mean (SD)	−0.97 (1.91)	–	−1.28 (2.07)	–		−6.61 (6.13)	–	−7.80 (6.37)	–
	Breath (%), mean (SD)	−1.00 (1.26)	–	−1.12 (1.16)	–		−4.01 (5.82)	–	−4.83 (6.19)	–
	Difference (%), mean (SD)	0.03 (0.49)	.95	−0.16 (0.59)	.79		−2.60 (1.80)	.16	−2.96 (2.22)	.19
	Cohen *d*	0.02	–	−0.10	–		−0.43	–	−0.47	–

^a^ITT: intent-to-treat.

### Feedback About Enhancement Strategies

Through open-ended questions in a web-based survey, we asked participants about their experiences with the different enhancement strategies. Some participants reported that the texts came at inconvenient times or were annoying. Others noted that the texts were very helpful and encouraging (eg, “I felt as though a friend was reminding me to stop rushing around, relax and be mindful”; “They give me occasional reminders that I am not on this journey alone”; and “They were good reminders to stay focused”). We asked participants who received the texts to rate how much they would recommend that we include them in the next study on a scale ranging from 1 (“don't include them, they were not helpful”) to 7 (“you must include them, they were very helpful”). On average, participants rated the texts as helpful (mean 5.36, SD 1.99). We asked participants to rate their overall satisfaction with the program on a scale ranging from 1 (“not at all satisfied”) to 7 (“very satisfied”). Both groups were satisfied with the program overall: those receiving the texts rated the program (mean 6.21, SD 0.89) and those not receiving the texts rated it (mean 6.12, SD 1.27; with a Cohen *d* of the difference between the groups of 0.08; *P*=.81).

In terms of the food and book gifts, participants reported that these helped them try new foods (eg, “...helped me to venture outside of my regular LCHF [low-carb, high-fat] menu”; “...let me try things first before spending lots of money on them”; “Very inspiring, and made trying new things possible”; and “OMGoodness these help soooooo much!”). We asked participants who received gifts to rate how much they would recommend that we include them in the next study on a scale ranging from 1 (“don't include them, they were not helpful”) to 7 (“you must include them, they were very helpful”). On average, participants rated the gifts as very helpful (mean 6.47, SD 1.30). Both groups were satisfied with the program overall: those receiving the gifts rated it (mean 6.40, SD 0.99) and those not receiving the gifts rated it (mean 5.94, SD 1.18; Cohen *d*=0.42; *P*=.25).

Some participants found the breath meter hard to use (eg, “I couldn't ever get it to work properly”; “I wanted the breath ketone meter [Ketonix] to work, but the readings are difficult to decipher”; and “I could never get the software to work on my computer [after several attempts]”). Others enjoyed using it (“I love the Ketonix! It's so easy to use and makes me aware of ketosis. I try to use it every day or at least 3 times a week now*.”*)

Participants did not make many comments about the Ketostix (urine strips), but one perceived it to be of potentially limited utility (“For me Ketostix indicators only showed small trace ketosis during my most successful weeks on the program so they don't really work well for me in terms of knowing if I'm successful or not on the program.”)

We asked participants to rate how much they would recommend that we include them in the next study from 1 (“don't include them, they were not helpful”) to 7 (“you must include them, they were very helpful”). On average, participants rated the ketone self-monitoring approaches as somewhat helpful: urine: mean 4.47 (SD 2.07); breath: mean 4.29 (SD 2.09; Cohen *d*=0.09, *P*=.82). Both groups were satisfied with the program overall: those receiving the urine strips rated it (mean 6.50, SD 0.73) and those receiving breath meter rated it (mean 5.80, SD 1.32; Cohen *d*=0.66; *P*=.07).

### Medication Changes

Although we intended to only recruit participants on no glucose-lowering medication (or only metformin), we erroneously randomized one participant who was taking sitagliptin. As metformin has a relatively low risk of hypoglycemia, physicians do not quickly reduce its dose. Therefore, as we intended to exclude participants on diabetes medications other than metformin, we had a limited ability to observe changes in glucose control medication. Overall glucose control medication changes (which were either for metformin or sitagliptin) included 3 discontinuations, 8 reductions (including the participant taking sitagliptin), 28 remaining the same, and 5 increases. Four participants were able to reduce their blood pressure medications, and 2 participants discontinued them.

### Other Health Changes

Self-reported adverse events that we considered attributable to the intervention included only minor complaints from a minority of participants, such as acne, constipation, nausea, and dizziness. Other self-reported adverse events that we do not believe are attributable to the intervention included one case each of cancer (skin and thyroid), injuries (back, knee, and shoulder), kidney stone, and surgeries (eye and herniated disc).

Many participants self-reported improvements in a variety of conditions or measures including low energy (“I have more energy now”), pain-related foot neuropathy (“Tingling, soreness, and pain have all gone away”), general pain (“No longer experience the almost daily body aches”), limited mobility (“Walking up stairs is not as grueling as it used to be”), headaches (“had frequent headaches [almost daily] which have completely resolved”), number of infections, allergic responses, acid reflux (several discontinued related medications), ability to focus their eyes, and high cholesterol and triglyceride levels.

## Discussion

The purpose of this study was to compare the addition of 3 intervention enhancement strategies (text messages, gifts, and urine vs breath ketone self-monitoring) that may help enhance the outcomes of a 12-month web-based *ad libitum* VLC diet and lifestyle intervention for adults with type 2 diabetes. Overall, among all participants, using ITT analyses, the mean HbA_1c_ was reduced by 1.0% and weight was reduced by 5.3%. Participants who completed the 12-month assessment reduced their mean HbA_1c_ by 1.2% and their weight by 6.3%. All these pre-post changes in mean HbA_1c_ and weight were statistically and clinically meaningful.

First, we examined the impact of sending intervention-relevant text messages to participants. Adding text messages to an intervention may add expense and complexity to the program, in addition to potentially increasing participant burden. However, among completers, the impact of text messages did meet our a priori threshold of Cohen *d* of 0.5 or greater for HbA_1c_ reduction. The mean differences in change in HbA_1c_ between those who received text messages and those who did not were 0.7% and 0.5% for completers and the full sample, respectively. This is similar to results from a previous meta-analysis of text message interventions used in patients with type 2 diabetes, which demonstrated a mean decrease in HbA_1c_ of 0.8% [11]. Moreover, our participants’ feedback suggested that they generally enjoyed the text messages and that they found them to be helpful. Future trials may benefit from sending participants intervention-relevant text messages.

Second, we tested the impact of mailing 6 rounds of gifts of diet-relevant foods and cookbooks. Providing gifts has been recommended as a strategy to enhance retention [43], and it may help with weight loss [12,13], but the use of food samples and cookbooks to assist with dietary changes is, to our knowledge, novel. Although the associated effect sizes of 0.2 to 0.3 fell short of our a priori threshold, participants rated the gifts positively and found them useful, and gifts may have improved participant enjoyment of the intervention (Cohen *d*=0.4). However, it is difficult to discern if these results were because of the fact that participants were receiving an incentive or if they were because of the impact of having these particular resources. Even so, if ample funding is available, then this strategy might benefit future trials.

Third, we assessed the impact of urine versus breath ketone self-monitoring. Self-monitoring may increase behavioral adherence [15,16] and can improve diabetes self-management [17] as well as weight loss and dietary adherence [18]. Yet, no previous trial, to our knowledge, has compared these 2 self-monitoring approaches. Among completers, the weight loss effect for urine ketone self-monitoring (vs breath ketone self-monitoring) met our a priori threshold. In contrast, several participants found the ketone breath meter difficult to use, and it is considerably more expensive than urine strips. Participants receiving the urine strips (vs the breath meter) may have enjoyed the program more overall (Cohen *d* of 0.7). Thus, future trials that include ketone testing may benefit from using urine-based rather than breath-based self-monitoring.

There are limitations to this study. The most notable limitation is the lack of statistical power to detect differences between the 2 levels of each of the 3 factors tested, because of the small sample size. This may have also reduced the stability of our estimates of effect sizes and changes. However, these preliminary results may provide insight into potentially effective methods for improving health outcomes in such a diet and lifestyle study.

### Conclusions

The results suggest that using text messages and urine-based ketone self-monitoring may be worthwhile enhancements for helping individuals with type 2 diabetes to adhere to a VLC diet intervention, which, in turn, is associated with reductions in HbA_1c_ and/or weight. In addition, diet-congruent food and cookbook gifts may improve participants’ overall intervention experience. Future research could investigate other enhancement strategies to help create even more effective approaches for treating type 2 diabetes.

## Abbreviations

HbA_1c_: glycated hemoglobin
IRB: institutional review board
ITT: intent-to-treat
NIH: National Institutes of Health
VLC: very low–carbohydrate, ketogenic diet
